# The Relationship Between State Boredom and Sleep–Wake Disruptions: A Mediation Model via Smartphone Addiction and Bedtime Procrastination

**DOI:** 10.3390/ijerph23060728

**Published:** 2026-05-30

**Authors:** Marco Fabbri, Monica Martoni

**Affiliations:** 1 Department of Psychology Renzo Canestrari, University of Bologna, 40127 Bologna, Italy; 2 Department of Medical and Surgical Sciences, University of Bologna, 40138 Bologna, Italy; monica.martoni@unibo.it

**Keywords:** boredom, smartphone addiction, bedtime procrastination, sleep quality, daytime sleepiness, sleep timing, mediation model

## Abstract

**Highlights:**

**Public health relevance—How does this work relate to a public health issue?**

- This study shows how state boredom affects sleep–wake quality and sleep timing.
- The influence of boredom on sleep–wake quality and sleep timing is mediated by smartphone addiction and bedtime procrastination.

**Public health significance—Why is this work of significance to public health?**

- This study proposes a mediation model linking several constructs relevant to public health, including boredom, smartphone addiction, bedtime procrastination, sleep quality, daytime sleepiness, and sleep timing.
- The model identifies multiple direct and indirect pathways among these variables, consistent with existing theoretical frameworks.

**Public health implications—What are the key implications or messages for practitioners, policy makers and/or researchers in public health?**

- Findings indicate that state boredom directly predicts sleep–wake problems, extending public health research beyond trait boredom (boredom proneness) and supporting cross-cultural investigation.
- The mediation model highlights potential intervention targets to improve sleep quality and daytime functioning.

**Abstract:**

Bedtime procrastination is linked to poor sleep quality, daytime sleepiness, and altered sleep timing. Identifying the factors influencing this behavior is crucial. Among them, problematic smartphone use can delay bedtime. State boredom, a multidimensional concept (high and low arousal, disengagement, inattention, and time perception), triggers problematic smartphone use as a way to cope with boredom, resulting in delayed bedtime and sleep–wake issues. This study aimed to test mediation models where state boredom predicts sleep-related outcomes both directly and indirectly through smartphone addiction and bedtime procrastination. A total of 259 participants (138 women; mean age = 38.44 years) completed an online survey, including the Mini-Sleep Questionnaire, Bedtime Procrastination Scale, Mobile Addiction Scale, Multidimensional State Boredom Scale, and measures of sleep timing on workdays and free days. Results showed significant positive associations among all variables. Mediation analyses revealed that state boredom directly predicted poor sleep quality and daytime sleepiness, and indirectly predicted smartphone addiction and bedtime procrastination. Additionally, boredom indirectly influenced sleep timing via bedtime procrastination. Overall, the findings suggest that boredom can lead to problematic smartphone use, which in turn delays bedtime, resulting in poorer sleep quality, increased daytime sleepiness, and delayed sleep timing.

## 1. Introduction

Sleep is a biological necessity in all mammals, and good sleep plays a crucial role in both physical and mental health. During sleep, the body is repaired and restored, memories are consolidated, and sleep contributes to overall functioning across different contexts, such as school, work, and leisure [1]. Sleep deprivation, insufficient sleep, or short sleep duration can negatively impact physical health (e.g., cardiovascular diseases [2]), mental health (e.g., contributing to depression and anxiety symptoms [3]), physiological balance and resilience [4], school performance [5], work efficiency [6], and sociability [7]. In addition, insufficient sleep can impair daytime functioning and reduce energy levels for the following day [8], while daytime activities and experiences can, in turn, influence subsequent nocturnal sleep [9]. The relationship between nighttime sleep and daytime functioning is explained by the two-process model of sleep regulation [10], which posits that the interaction between a homeostatic process (S) and a circadian process (C) regulates the sleep–wake cycle. Daytime fatigue and sleepiness have been shown to impair cognitive functioning, leading to lower academic performance among students [11], reduced productivity, and increased susceptibility to errors and accidents in occupational settings [12]. In modern society, disturbances in the sleep–wake cycle can negatively affect health, quality of life, and productivity [13]. Therefore, it is important to investigate the factors contributing to good sleep–wake quality.

Among these factors, bedtime procrastination, defined as going to bed later than intended without external reasons for the delay [14], has received increasing attention. Considering that procrastination is defined as the “*voluntary delay of an intended course of action despite expecting to be worse off for the delay*” ([15], p. 66), bedtime procrastination represents a specific form of procrastination related to sleep behavior [16]. Its relevance in the health domain stems from the fact that going to bed late is associated with insufficient sleep duration, which in turn is linked to adverse outcomes such as cognitive impairments (e.g., memory errors [17]) and physical diseases (e.g., cardiovascular conditions [18]). Unlike general procrastination, which typically involves delaying aversive tasks [15], bedtime procrastination does not necessarily involve aversive activities. Instead, individuals may actively choose to stay awake (e.g., reading in bed) or passively delay sleep (e.g., scrolling through social media or watching TV without awareness of time passing) [19]. Kroese et al. [14] demonstrated that bedtime procrastination predicts sleep duration, daytime fatigue, and insufficient sleep beyond the effects of demographic variables and self-regulation. Furthermore, bedtime procrastination has been associated with poor sleep quality [20], wake-up times [21], later dinner times [22], bedtime routines and immersive activities (e.g., watching TV or using smartphones) [23], feelings of sleep deprivation and daytime fatigue [24,25], and biological factors such as chronotype [26]. Hill et al. [25], in a systematic review and meta-analysis, reported that bedtime procrastination is primarily associated with eveningness, shorter sleep duration, poorer sleep quality, and increased daytime fatigue, suggesting its impact on sleep–wake rhythmicity and behavior. However, bedtime procrastination may affect sleep–wake patterns differently on workdays versus weekends, as sleep schedules often vary between these periods [27,28]. For example, studies in adolescents and young adults have found that procrastination is associated with shorter sleep duration on weekdays but not on weekends [29,30]. These findings suggest that bedtime procrastination may be more prevalent during demanding days (e.g., school or workdays), when individuals delay sleep to complete postponed tasks or engage in leisure activities, whereas it may be less pronounced on free days. However, evidence in the general population remains limited, and the present study aims to address this gap.

The widespread use of electronic devices may contribute to bedtime procrastination. Modern smartphones offer numerous functionalities, enabling communication, access to information, navigation, and health-related support [31]. However, excessive and prolonged smartphone use has been associated with physical, mental, and behavioral problems [32], leading to increasing attention to smartphone addiction [33]. Smartphone addiction is considered a behavioral addiction characterized by excessive and uncontrolled use, withdrawal symptoms, and functional impairment [34]. Several studies have reported negative effects of screen time on sleep quality and quantity [35,36,37]. Smartphone overuse has also been associated with daytime tiredness, longer sleep latency, and reduced sleep duration [38,39,40]. In particular, smartphone use close to bedtime can delay circadian rhythms, negatively affecting both sleep duration and quality [41]. Moreover, excessive smartphone use has been linked to increased psychological stress and arousal, which can further impair sleep and recovery [31]. For instance, studies on workaholics have shown bidirectional relationships between smartphone use, poor sleep quality, and daytime sleepiness [42]. Importantly, the relationship between problematic smartphone uses and poor sleep quality appears to be mediated by bedtime procrastination [43]. High levels of smartphone use, and bedtime procrastination may lead to shorter sleep duration, difficulty falling asleep, and insomnia [44,45], possibly due to the design of mobile applications, which are intended to maintain user engagement and increase pre-sleep arousal [46,47]. Thus, individuals with higher levels of smartphone addiction may postpone sleep-related activities in favor of engaging with their devices, seeking gratification or relaxation after daily activities, thereby contributing to bedtime procrastination [43,48,49,50,51,52].

Boredom may represent an important factor underlying problematic smartphone use [53,54]. It is defined as a tendency to experience a lack of interest, meaning, excitement, and challenge [55]. Individuals may cope with boredom by engaging in stimulating online activities [56,57,58]. At the same time, it has been reported that students with perceived high stress were also more dependent on their smartphones [59]. However, smartphone use to cope stress was associated with smartphone addiction, and this smartphone dependency was associated with less effective coping strategies, such as avoidance [59]. Moreover, longitudinal research has shown that boredom proneness predicts increases in smartphone addiction over time [60,61], suggesting that reducing boredom could help mitigate problematic smartphone use. Boredom has also been associated with poor sleep quality, as individuals may resist going to bed in search of more engaging activities [62,63,64,65]. Recent studies, including those conducted during the COVID-19 pandemic, have linked boredom and altered time perception to poorer sleep quality and delayed bedtime [66,67,68,69,70,71]. Furthermore, poor sleep may reinforce feelings of boredom, creating a bidirectional relationship [72,73,74]. Boredom has also been shown to predict inattention, which in turn is associated with bedtime procrastination and poor sleep quality [75,76,77]. These findings suggest that boredom may drive individuals toward smartphone use as a means of stimulation, reinforcing problematic usage patterns [78,79]. This, in turn, may increase pre-sleep arousal and contribute to bedtime procrastination [80,81]. Importantly, previous studies have primarily focused on trait boredom (i.e., a general tendency to experience boredom), whereas less attention has been paid to state boredom, defined as the situational experience of boredom at a given time [82]. Assessing state boredom allows for testing temporal relationships and potential causal mechanisms linking boredom to sleep–wake outcomes. For example, Fahlman et al. [82] have developed a full-scale measure of state boredom to assess the individual’s experience of boredom in a specific moment (e.g., “*I feel bored now*” or “*I felt bored yesterday*”) according to theoretical definition of boredom. This multidimensional definition of boredom is based on five dimensions, such as high and low arousal, disengagement, inattention and time perception [82]. Moreover, not only Fahlman et al. [82] demonstrated that this multidimension definition of the state boredom significantly correlated with trait boredom, but also, they showed significant associations between state boredom and depression and life satisfaction which both have been related to problematic mobile use, bedtime procrastination, sleep quality and daytime sleepiness [83,84,85]. Thus, it is possible to assume that people who experience boredom in a specific moment tend to cope this situation in using their mobile phones, procrastinating their bedtime, inducing poor sleep quality and high daytime sleepiness. These assumptions could be based, not only, on the relationships between trait boredom and smartphone addiction, bedtime procrastination, and sleep–wake problems due to the link between trait and state boredom constructs, but also, on the association between each component of state boredom with bedtime procrastination [52,71,75,76,77,79] and problematic mobile phone use [86,87,88,89], suggesting in this latter case that the smartphone use serves as a “*safety behaviour*” or emotional strategies for regulating boredom and distress.

**Figure 1:**
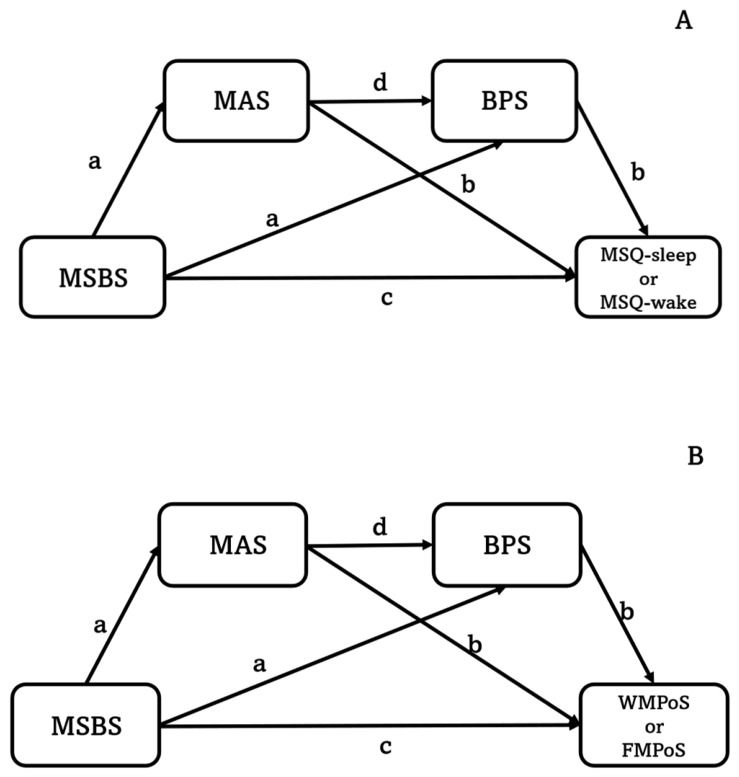
(**A**) The mediation model illustrates the direct effect of state boredom (MSBS) on poor sleep quality (MSQ-sleep) and daytime sleepiness (MSQ-wake), as well as indirect effects through smartphone addiction (MAS) and/or bedtime procrastination (BPS); (**B**) The mediation model illustrates the direct effect of state boredom (MSBS) on the midpoint of sleep during weekdays (WMPoS) and free days (FMPoS), as well as indirect effects through smartphone addiction (MAS) and/or bedtime procrastination (BPS). In both figures, c indicates the direct effect, while a, b, and d indicate the indirect effects and the different pathways tested.

The present study aimed to examine the relationships among boredom, smartphone addiction, bedtime procrastination, and sleep–wake quality in adults. Based on the literature, higher levels of boredom were expected to be associated with greater smartphone addiction, higher bedtime procrastination, and poorer sleep quality and daytime functioning. We tested mediation models (Figure 1) to assess whether state boredom predicts sleep problems and daytime sleepiness both directly and indirectly through smartphone addiction and bedtime procrastination. Additionally, we examined whether these relationships extend to sleep timing (midpoint of sleep [90]) on workdays and weekends, hypothesizing that these effects would be more pronounced on workdays [29,30].

## 2. Materials and Methods

### 2.1. Participants

In this online survey, we recruited 259 participants (138 women and 121 men) aged between 18 and 76 years (M = 38.44 years, SD = 15.27). All participants took part voluntarily, anonymously, and without compensation, with the option to withdraw at any time. In the sample, 54.40% of participants held a university degree (bachelor’s or master’s), 28.20% had a high school diploma, 7.40% had a middle or elementary school diploma, and 3.50% had a professional school diploma. The remaining 6.60% held a PhD and/or a postgraduate specialization degree. Overall, 73.70% of participants reported being employed, with the majority (about 60%) having a full-time contract and approximately 20% a part-time contract. In addition, 12% reported on-call work contracts, while the remaining participants reported other forms of employment. Regarding marital status, 139 participants were single, 109 were married or cohabiting with a partner, 10 were divorced, and one was widowed. Participants were recruited through university courses, social media, and snowball sampling techniques (e.g., word of mouth).

The study protocol was approved by the Ethics Committee of the Department of Psychology at the University of Campania Luigi Vanvitelli, where the corresponding author was affiliated at the time of the study. All participants provided informed consent through four separate consent forms (by clicking four times the “Agree” or “Consent” button after carefully reading the informed consent, privacy, and data management statements). Participants who did not agree clicked the “Disagree” or “Do not consent” button and exited the survey.

### 2.2. Materials

#### 2.2.1. Mini-Sleep Questionnaire (MSQ)

A 10-item scale developed by Natale et al. [91] was used to assess the frequency of sleep–wake-related behaviors over the past two weeks. The Italian version of the MSQ was adopted [91], and responses were recorded on a 7-point Likert scale ranging from 1 (never) to 7 (always). Total scores indicated overall sleep–wake problems, with higher scores reflecting greater impairment. The MSQ comprises two subscales: sleep (5 items; e.g., *“Difficulty falling asleep”*) and wake (4 items; e.g., *“Excessive daytime sleepiness/falling asleep during the day”*). Item 6 (snoring) did not load on any factor. Higher scores on each subscale indicated greater dysfunction. Based on Natale et al. [91], a score >16 on the sleep factor indicated poor sleep quality/sleep problems, whereas a score >14 on the wake factor indicated excessive daytime sleepiness. Internal consistency was Cronbach’s α = 0.87 for the total scale, α = 0.80 for the sleep subscale, and α = 0.81 for the wake subscale.

#### 2.2.2. Midpoint of Sleep (MPoS)

Four ad hoc questions [92,93,94] asked participants to indicate their usual bedtime (BT) and wake-up time (WT) during workdays (W) or university days (typically Monday to Friday), and free (F) days (typically weekends). These data allowed calculation, in hours:minutes, of the midpoint of sleep (MPoS), defined as the midpoint between bedtime and wake-up time for working days (WMPoS) and free days (FMPoS).

#### 2.2.3. Bedtime Procrastination Scale (BPS)

A 9-item scale developed by Kroese et al. [14] was used to assess bedtime procrastination (e.g., *“I go to bed early if I have to get up early in the morning”*). To the best of our knowledge, no Italian version was available; therefore, we developed one using a back-translation procedure with a native English speaker. Discrepancies were identified and resolved iteratively until equivalence was achieved. Items were rated on a 5-point scale from 1 (never) to 5 (always). After reversing four items, total scores ranged from 9 to 45, with higher scores indicating greater bedtime procrastination. Reliability in the present study was Cronbach’s α = 0.85.

#### 2.2.4. Mobile Addiction Scale (MAS)

A 13-item scale developed by Fridan [95] was used to assess mobile addiction within the framework of behavioral addiction [96,97,98,99,100]. An Italian version was created using the same back-translation procedure described above. Items were rated on a 5-point scale from 1 (almost never) to 5 (almost always). The MAS includes several components: Salience (2 items; e.g., *“Things that keep me from using mobile phones are boring to me”*), Tolerance (3 items; e.g., *“I feel I need to recheck soon after using the mobile phone”*), Withdrawal (2 items; e.g., *“Life is empty without my mobile phone”*), and Relapse (3 items; e.g., *“I reduce my mobile phone usage, but it increases again”*). Additionally, three items assess Internet use via mobile phone (e.g., *“I use programs on my mobile phone that require the Internet”*). These components were combined using the following formula:

*Mobile Addiction score* = 2.637 + (0.625 × Tolerance) + (0.444 × Relapse) + (0.295 × Salience) + (0.44 × Withdrawal) + (0.475 × Internet) (1)

Higher values indicate greater mobile addiction. Although the original study also identified a Conflict component (3 items; e.g., *“Using my mobile phone in a circle of friends has been seen as a problem”* [96]), it was not significantly associated with mobile addiction and was therefore excluded. Reliability in the present study was Cronbach’s α = 0.90.

#### 2.2.5. Multidimensional State Boredom Scale (MSBS)

A 29-item scale developed by Fahlman et al. [82] was used to assess the momentary experience of boredom. The Italian version was adopted and has shown good psychometric properties [101]. Items were rated on a 7-point Likert scale from 1 (strongly disagree) to 7 (strongly agree). Total scores reflect overall state boredom, with higher scores indicating greater boredom. The scale includes five factors: Disengagement (10 items; e.g., *“I am stuck in a situation that I feel is irrelevant”*), High Arousal (5 items; e.g., *“Everything seems to be irritating me right now”*), Low Arousal (5 items; e.g., *“I feel down”*), Inattention (4 items; e.g., *“I am easily distracted”*), and Time Perception (5 items; e.g., *“Time is passing by slower than usual”*). Reliability for the total scale was Cronbach’s α = 0.96; for the subscales, α values ranged from 0.89 to 0.91.

### 2.3. Procedure

This study was conducted as an online survey using the PsyToolkit platform [102,103]. The survey was disseminated through undergraduate and master’s psychology courses (i.e., the study was presented during bachelor’s and master’s courses) and major social media platforms (Facebook, Instagram, X ex Twitter, and WhatsApp). Interested participants received a link to the survey and were invited to share it with acquaintances (snowball sampling). Specifically, each participant was invited to share the online link to parents, friends, colleagues, etc., or to explain them how to contact the researchers to receive the link of the study. Inclusion criteria were being of legal age and proficient in Italian. The survey began with a study description and instructions for completion. After providing informed consent, participants completed socio-demographic questions followed by MSQ, BPS, MAS, and MSBS. A debriefing and contact information were provided at the end.

### 2.4. Data Analysis

Analyses were conducted using SPSS Statistics version 20 (IBM Corporation, New York, United States). First, descriptive statistics (means and standard deviations) were calculated, and associations between socio-demographic variables and study variables were examined. Second, partial correlations among study variables were computed, controlling for gender, age, education, and marital status. Third, mediation analyses were performed using the same covariates to test whether smartphone addiction (MAS) and bedtime procrastination (BPS) mediated the relationships between state boredom (MSBS) and poor sleep quality (MSQ-sleep), daytime sleepiness (MSQ-wake), and midpoint of sleep during working days (WMPoS) and free days (FMPoS). Mediation analyses were conducted using the PROCESS macro (Model 6) developed by Hayes [104]. Indirect effects were tested using bootstrapping with 5000 resamples and 95% bias-corrected confidence intervals (95% CIs) [105]. A conservative alpha level of 0.01 was adopted to account for multiple comparisons [71,106].

## 3. Results

Table 1 summarizes the descriptive statistics of all variables, along with their associations with socio-demographic characteristics. Based on the selected alpha level, women reported higher levels of subjective wake-related problems than men (*t*(257) = −3.53, *p* = 0.0001). Additionally, younger individuals reported higher MSQ-wake scores (*r* = −0.35, *p* = 0.0001). Age was negatively associated not only with both MPoS scores (WMPoS: *r* = −0.35, *p* = 0.0001; FMPoS: *r* = −0.55, *p* = 0.0001) but also with MSBS and MAS scores (both correlations were *r* = −0.26, *p* = 0.0001).

Regarding educational level, only the midpoint of sleep during working days (WMPoS) was negatively associated with lower educational attainment (*rho* = −0.25, *p* = 0.0001). Occupational status was not significantly associated with any of the variables. In contrast, marital status showed significant effects: single participants reported higher MSQ-wake (*F*(2,256) = 7.79, *p* = 0.0001), MSBS (*F*(2,256) = 18.29, *p* = 0.0001), and MAS scores (*F*(2,256) = 7.22, *p* = 0.001), as well as later WMPoS (*F*(2,256) = 17.58, *p* = 0.0001) and FMPoS (*F*(2,256) = 45.35, *p* = 0.0001), compared to participants who were previously married, with those cohabiting with a partner showing intermediate values. Accordingly, gender, age, education level, and marital status were included as covariates in subsequent analyses.

Table 2 shows that all variables were positively correlated, indicating that state boredom (including its subcomponents), smartphone addiction, bedtime procrastination, sleep–wake problems, and the midpoint of sleep during both workdays and weekends are interrelated (*r* values ranged from +0.17 to +0.53). Notably, two distinct patterns emerged: WMPoS was positively associated with MSQ-wake (*r* = +0.20, *p* = 0.001), high arousal (*r* = +0.20, *p* = 0.001), inattention (*r* = +0.25, *p* = 0.0001), MSBS (*r* = +0.18, *p* = 0.0001), MAS (*r* = +0.17, *p* = 0.0001), and BPS (*r* = +0.20, *p* = 0.001), whereas FMPoS was positively associated only with inattention and BPS. Furthermore, no significant correlation was found between BPS (*r* = +0.30, *p* = 0.0001) and the inattention dimension (*r* = +0.17, *p* = 0.0001) of the MSBS.

**Figure 2:**
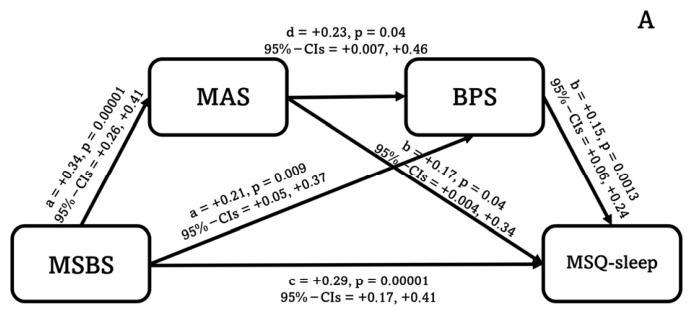

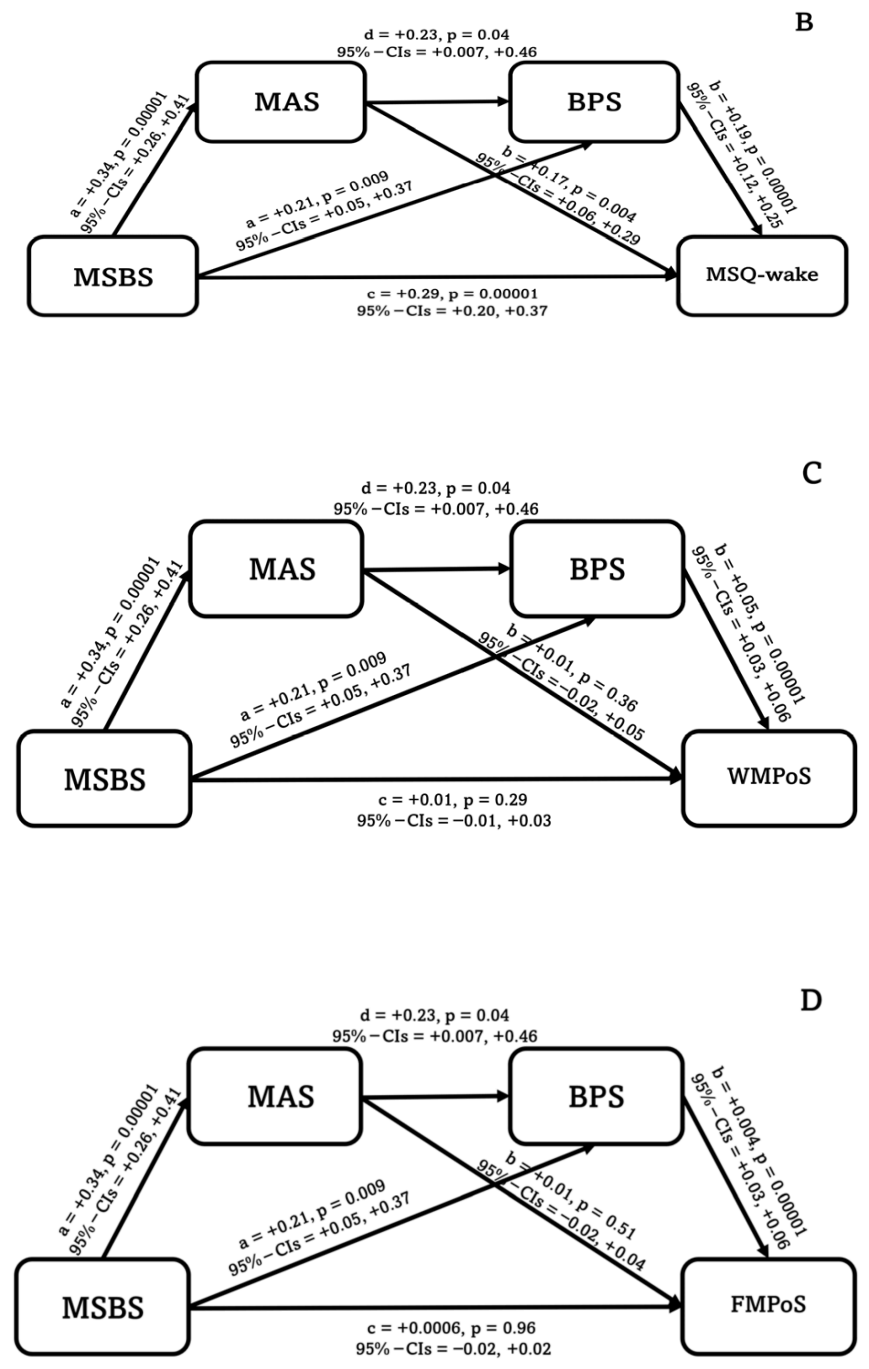
The model illustrates the role of state boredom (MSBS) in predicting (**A**) poor sleep quality (MSQ-sleep), (**B**) daytime sleepiness (MSQ-wake), (**C**) midpoint of sleep during working days (WMPoS), and (**D**) midpoint of sleep during free days (FMPoS), both directly and indirectly through smartphone addiction (MAS) and bedtime procrastination (BPS).

Table 3 summarizes the direct and indirect effects in each mediation model. The mediation model predicting the MSQ-sleep factor was statistically significant (*R*^2^ = 0.19, *F*(5,253) = 11.71, *p* = 0.00001), as shown in Figure 2A. We observed both a direct effect (Table 3), indicating that the situational boredom was related to poor sleep quality and two indirect effects: the first path illustrated how state boredom was associated with high bedtime procrastination, which, in turn, was related to poor sleep quality (Table 3) and the second path displayed that high situational boredom was linked to elevated smartphone addiction, which, in turn, was associated with high bedtime procrastination, ultimately predicting poor sleep quality (Table 3). The indirect pathway linking MSBS to MSQ-sleep through MAS alone was not significant (Table 3). Overall, this first mediation model (Figure 2A) confirmed, on one hand, the relationship between state boredom and poor sleep quality, while, on the other hand, it demonstrated the role of bedtime procrastination in this relationship. Importantly, we also found that individuals coped with situational boredom by using their smartphones, which delayed bedtime and negatively impacted sleep quality.

Similar patterns were observed in the models predicting WMPoS (*R*^2^ = 0.20, *F*(5,253) = 12.60, *p* = 0.00001) and FMPoS (*R*^2^ = 0.34, *F*(5,253) = 26.15, *p* = 0.00001), as illustrated in Figure 2C,D. In both models, indeed, high levels of boredom were related to high BPS scores, which, in turn, predicted a delayed sleep timing, as well as situational boredom was associated with high smartphone addiction, which was, then, related to high BPS score, ultimately predicting sleep timing (Table 3). In addition, we did not find any direct effect of state boredom on sleep timing during working and free days (Table 3). As before, the pathway linking state boredom to sleep timing through MAS score was not significant (Table 3). The mediation models shown in Figure 2C,D largely mirrored the findings of the previous model, confirming that problematic mobile phone use served as a maladaptive coping strategy for state boredom, leading to increased bedtime procrastination and, consequently, delayed sleep onset during the week.

The mediation model predicting the MSQ-wake factor was also statistically significant (*R*^2^ = 0.39, *F*(5,253) = 32.91, *p* = 0.00001), as shown in Figure 2B. In this case, MSBS score predicted daytime sleepiness (Table 3). Also, high situational boredom predicted daytime sleepiness both through elevated MAS and high BPS scores (Table 3). Finally, we found that high levels of state boredom were associated with elevated MAS scores, which in turn were linked to increased bedtime procrastination, ultimately predicting higher daytime sleepiness (Table 3). This mediation model, not only, mirrored the previous findings, but also, displayed the indirect involvement of MAS score in the relationship between state boredom and daytime sleepiness, suggesting that the boredom experience induced a problematic mobile phone usage, which could determine daytime sleepiness.

## 4. Discussion

The aim of the present study was to examine the relationship between state boredom, smartphone addiction, bedtime procrastination, and sleep–wake quality in a sample of adults. In addition, we explored whether these variables were associated with sleep timing, defined as the mid-point of sleep on workdays and free days [29,30,71,92,93].

This online survey revealed specific associations between sleep–wake disturbances and delayed sleep timing with multidimensional state boredom, smartphone addiction, and bedtime procrastination. Indeed, in this cross-sectional study, we found that higher levels of boredom, greater problematic smartphone use, and a stronger tendency to intentionally delay bedtime were associated with poor sleep quality, increased daytime sleepiness, and later sleep timing on both workdays and free days. The correlation analyses confirmed that poor sleep quality and daytime sleepiness were positively associated with bedtime procrastination [14,16,17,20,21,23,24,25]. Bedtime procrastination has been linked to sleep deprivation, impaired cognitive performance, and reduced productivity or academic performance [25]. Given that we administered the MAS within the framework of mobile addiction [95,96,97,98,99,100], our positive correlations support the negative impact of screen time on sleep quality and quantity, daytime fatigue, longer sleep latency, and reduced sleep duration [35,38,39,40]. Specifically, evening smartphone use may delay circadian rhythms, as light-emitting devices impair alertness the following morning [42,107,108,109]. Finally, we found positive correlations between sleep–wake problems and each component of boredom, as well as the total MSBS score. Sleep–wake disturbances were thus associated with the aversive experience of an unfulfilled desire for engagement in satisfying activity, with altered levels of arousal, with the perception of slow passage of time, with inattention, and, overall, with state boredom. Considering the association between trait and state boredom [82], these findings extend the previous literature on the relationship between the sleep–wake cycle and boredom proneness (trait boredom) [44,45,62,63,64], using a broader conceptualization of boredom. Additionally, our results align with findings from real-world experiences of boredom, such as during the COVID-19 lockdown [110], which induced sleep–wake disturbances through daily rhythm dysregulation [66,67,68,69,70,71,72,73].

Mediation models provided a possible explanation of how these variables were related to each other. Regarding sleep–wake problems (Figure 2A,B), state boredom seemed to directly and indirectly predict poor sleep quality and daytime sleepiness. The first indirect pathway showed that the experience of feeling bored in a specific moment was positively associated with bedtime procrastination, which, in turn, may predict poor sleep quality and daytime sleepiness. A possible explanation of this pathway could be related to the failure of self-regulation [14,15,16] in attempting to cope with negative feelings associated with boredom. Considering that bedtime procrastination is a type of failure of self-regulation, this indirect pathway could indicate that poor sleep quality and daytime sleepiness were associated with the delay in going to bed deliberately due to an alteration of self-regulation in situational boredom [111,112,113]. In other words, bored people have urge to get rid of boredom with the search of something interesting to do. When this urge is near bedtime, then it might contribute to bedtime procrastination [75,76,77,78,79,80], due to the continuous search for something interesting. The second indirect pathway could indicate that boredom was positively associated with smartphone addiction, which, in turn, predicted poor sleep quality and daytime sleepiness. In this case, this pathway could suggest that situational boredom drives individuals to escape aversive states, and excessive smartphone use may serve as a daily strategy to alleviate multidimensional boredom, as well as the negative feeling associated with this state boredom. Such overuse, particularly at bedtime, may delay, replace, or disrupt bedtime routines, induce higher psychological arousal, and negatively impact sleep [31]. Additionally, blue light exposure from screens may interfere with melatonin production, further affecting wakefulness [31]. Although the MAS is a measure of smartphone addiction, this second mediation model may indicate that a daily strategy to cope with boredom when it occurs is to (over-)use the smartphone, which helps people to cope with distress and boredom [59,86,87,88,89]. However, the excessive (or problematic) mobile phone use may disrupt sleep quality via circadian misalignment due to light-emitting devices and activate neurophysiological systems that promote wakefulness, counteracting the homeostatic sleep drive and further disrupting the sleep–wake cycle [114,115,116,117]. Moreover, excessive smartphone use before bedtime may contribute to sleep disturbance by increasing arousal levels due to the interactive nature of the devices which elevates mental alertness [118], in individuals with altered levels of arousal, which is one of component of boredom. Notably, in the mediation model with MSQ-wake factor (Figure 2B), an additional indirect effect emerged: state boredom was associated with smartphone addiction, which in turn was related to daytime sleepiness. This suggests that individuals experiencing boredom may use smartphones to self-stimulate, maintain attention, or cope with lethargy and agitation, ultimately leading to persistent sleepiness, low energy, and daytime fatigue. The absence of this indirect pathway for the MSQ-sleep factor may be related to the arousal levels associated with boredom, as well as the frequent use of smartphones to cope with state boredom, in similar way to what has been reported regarding the impact of video games on sleep [119]. Further studies are needed to investigate smartphone use as a coping strategy to better explain the relationship between state boredom and sleep–wake disturbances. Regarding sleep timing, defined as mid-point of sleep [92,93,117], these patterns were largely replicated, except for the lack of a direct effect of boredom. Positive correlations were observed between bedtime procrastination and mid-point of sleep on both workdays and weekends, in contrast to findings in adolescents [29,30]. This discrepancy may reflect sample differences, as adults are likely better able to accommodate social and environmental demands, such as work or academic schedules, during weekdays. Correlation analyses revealed distinct patterns for WMPoS and FMPoS: WMPoS correlated positively with high arousal, inattention, MSBS total score, and MAS, whereas FMPoS correlated only with inattention. This suggests that on workdays, stress, distraction, arousal, boredom, and problematic smartphone use may increase bedtime procrastination. In this case, the impact of social, environmental, and daily activities and stressors may contribute to boredom experience, smartphone addiction, and bedtime procrastination, impacting the sleep timing. For example, our data could be in line with those reported by Spagnoli et al. [42], who reported the relationship between smartphone use and sleep timing through the mediation role of workaholism. On the opposite, the different pattern of associations during free days could be related to the high probability of experiencing relaxing feelings when there is lower stress and social (work or school) demands. Mediation models (Figure 2C,D) mirrored the pathways in Figure 2A, indicating that boredom, smartphone addiction, and bedtime procrastination interactively influence sleep timing. Bored individuals, unable to engage in meaningful activity, may seek stimulation via smartphones, which distracts from attention to the present moment and delays bedtime, ultimately shifting sleep timing and affecting sleep health [20,69,70,71,72,73,74,75,77,117].

Overall, our findings may have important public health implications in modern societies, where irregular sleep is prevalent due to chronic circadian disruption [120]. In our opinion, our mediation models could provide a theoretical foundation for practical interventions in public health. First, the study underscores the importance of maintaining a regular sleep–wake cycle and consistent sleep timing for sleep hygiene and health, as well as for sleep medicine and psychology [71,117,120]. Second, interventions targeting general and bedtime-specific procrastination may improve sleep quality and daytime functioning [121]. Effective time management and procrastination reduction can enhance sleep duration and quality, reduce daytime fatigue, and increase energy and efficiency in daily activities. For instance, promoting and maintaining a structured routine may be associated with lower levels of procrastination [121]. A possible intervention could focus on self-regulation or self-control, such as cognitive and behavioural training [122] to contribute to ameliorate health outcomes associated with a wide variety of diseases and disorders. Linked to this aspect, a third targeting intervention is related to support strategies for preventing smartphone addiction, including behavioural interventions to regulate usage and promote media literacy [123], as well as promoting physical activities and reducing sedentary behaviour. Given that individuals with lower levels of general self-control tend to have reduced planning abilities and greater difficulty resisting temptations [113], an intervention aimed at enhancing self-control could improve the ability to inhibit automatic responses and promote more mindful smartphone use. Interventions could also encourage physical activity (and reduce sedentary behaviours), more time spent in natural settings (e.g., urban park [76]), and face-to-face social interactions rather than excessive technology-mediated engagement. Fourth, Mindfulness-Based Interventions may help reduce boredom, enhance present-moment attention, decrease bedtime procrastination, and positively affect sleep quality and timing [71,75,76,77,124,125]. These findings provide potential strategies for coping with boredom and maintaining regular sleep–wake cycles in industrialized societies. Future studies should investigate the implementation of targeted daily interventions, particularly in at-risk populations such as adolescents [126]. For example, academic boredom has been identified as a negative emotional experience, associated with a range of problematic academic outcomes, including anger, anxiety, and shame, as well as reduced motivation and effort, lower academic achievement, and higher dropout rates [126]. Similarly, boring or monotonous work environments are linked to both emotional and physical health issues, as well as stress-related health problems [127]. The present study extends this by examining the impact of boredom, through smartphone addiction and bedtime procrastination, on public health concerns related to poor sleep quality and daytime sleepiness. These issues affect physical and mental health [1,2,3,4], school performance [5,11], work efficiency and productivity [6,12,13], sociability [7,8], and, overall, quality of life [13].

This study has several limitations. First, although the questionnaires had good psychometric properties, self-reported measures are subject to bias. Future studies should consider objective methods, such as experimental manipulation of boredom (e.g., a sensory deprivation condition), smartphone usage tracking (e.g., app for measuring the duration and frequency of smartphone usage), and actigraphic sleep–wake monitoring or accelerometers to assess physical activity and/or to examine lifestyle. Second, the convenience sampling and the data collection approach limit the generalizability of our findings, despite controlling for socio-demographic variables. These methodological aspects could limit the external validity of the study. For instance, individuals who experience boredom more frequently or have problematic mobile phone use may have been more likely to participate in the survey, which could reduce the representativeness of the sample. Also, our sample was unbalanced for education level, given that most participants were recruited in university settings, limiting representativity. In addition, in our sample, we included a wide range of participants from 18 years to 76 years. Although we inserted age as covariate in all analyses performed, age group differences were not analyzed and this limits the interpretability of the findings, as potential developmental or cohort effects are not accounted for. Future studies should promote the survey with probabilistic sampling methods with the control of cohort effect. Third, the cross-sectional design precludes causal inferences regarding the effects of boredom on sleep–wake patterns and timing via smartphone addiction and bedtime procrastination. Longitudinal or experimental studies are needed to examine causal relationships. Related to this point, we proposed a theoretical model in which, basically, boredom predicted directly and indirectly sleep–wake problems and sleep timing. However, alternative models could be highlighted. For example, poor sleep quality and daytime sleepiness reinforced feelings of boredom, and, consequently, the adoption of “safety strategies” to contrast these negative feelings and/or delaying the moment of going to bed, contributing to delayed sleep–wake patterns the following day [66,67,68,69,70,71,72,73,74,75], likely forming a self-perpetuating cycle. Finally, correlation analyses revealed small-to-moderate effect sizes, and mediation models showed direct and indirect effects with small-to-moderate strengths, as well as low *R*^2^ values. Although we applied a conservative *p*-value to account for multiple comparisons, future research should replicate these findings in larger samples.

## 5. Conclusions

The aim of the present study was to examine the relationship between boredom, mobile phone addiction, bedtime procrastination, and sleep–wake quality in a sample of adults. In addition to the positive correlations found among all variables, the main results revealed that state boredom, defined multidimensionally, predicted both directly and indirectly poor sleep quality, daytime sleepiness, and sleep timing. Two indirect pathways were consistently identified: when individuals experienced boredom, they were more likely to use their smartphones problematically as a way to cope with these negative emotions, which in turn negatively affected sleep–wake patterns and sleep timing; on the other hand, when people experienced situational boredom, it led them to seek stimulating activities or something interesting to do, delaying their bedtime. The novelty of the present study lies in proposing a theoretical model that links state boredom, problematic mobile phone use, bedtime procrastination, and sleep–wake patterns, extending the previous data due to the association between trait and state boredom [82]. This model emphasizes its relevance to individual health and well-being across various settings, such as academic and occupational environments.

## Figures and Tables

**Table 1:** Means and standard deviations (SDs) for all variables are reported. The table also presents means (and SDs) for men (M), women (F), employed participants (Work), unemployed participants (No Work), and marital status groups, along with the corresponding statistical values (*t*, *p*, and *Cohen’s d*, or *F*, *p*, and partial eta-squared or *ƞ*^2^*_p_*). Pearson’s correlation coefficients (*r*) or Spearman’s correlation coefficients (*rho*), with corresponding *p*-values, are also reported. Significant results are indicated in bold.

	Descriptive	Gender					Age	Education	Occupational Status					Marital Status					
	M (SD)	M (*n* = 121)	F (n = 138)	*t*(257)	*p*	*Cohen’s d*	*r* (and *p*)	*rho* (and *p*)	Work (n = 191)	No Work (n = 68)	*t*(257)	*p*	*Cohen’s d*	Single	With Partner	Previously Married	*F*(2,256)	*p*	*Ƞ* ^2^ *_p_*
**MSQ-sleep**	16.14 (5.94)	15.73 (5.99)	16.50 (5.90)	−1.04	0.30	0.13	−0.06 (*p* = 0.34)	−0.06 (*p* = 0.31)	16.24 (6.19)	15.87 (5.22)	0.44	0.66	−0.062	16.47 (6.28)	16.01 (5.63)	1318 (3.74)	1.62	0.20	0.01
**MSQ-wake**	13.93 (5.11)	**12.76 (5.05)**	**14.96 (4.95)**	**−3.53**	**0.0001**	**0.44**	**−0.35** **(*p* = 0.0001)**	−0.09 (*p* = 0.14)	14.14 (4.95)	13.35 (5.53)	1.09	0.28	−0.16	**15.04 (5.03)**	**12.78 (4.95)**	**11.36 (4.63)**	**7.79**	**0.0001**	**0.06**
**WMPoS**	03:32 (01:08)	03:30 (01:16)	03:34 (01:01)	−0.45	0.65	0.05	**−0.35** **(*p* = 0.0001)**	**−0.25** **(*p* = 0.0001)**	03:26 (01:08)	03:49 (01:06	−2.40	0.017	0.34	**03:54 (01:13)**	**03:07 (00:53)**	**02:59 (00:42)**	**17.58**	**0.0001**	**0.12**
**FMPoS**	04:48 (01:20)	04:35 (01:22)	04:59 (01:17)	−2.39	0.018	0.30	**−0.55** **(*p* = 0.0001)**	−0.13 (*p* = 0.034)	04:44 (01:19)	04:59 (01:25)	−1.14	0.18	0.19	**05:26 (01:18)**	**04:04 (00:56)**	**04:01 (01:14)**	**45.35**	**0.0001**	**0.26**
**MSBS**	17.75 (6.66)	16.92 (7.42)	18.48 (5.84)	−1.90	0.06	0.24	**−0.26** **(*p* = 0.0001)**	−0.02 (*p* = 0.77)	17.50 (6.69)	18.45 (6.56)	1.01	0.31	0.14	**19.89 (6.34)**	**15.47 (6.20)**	**13.21 (5.59)**	**18.29**	**0.0001**	**0.13**
**MAS**	18.12 (4.59)	18.05 (4.80)	18.18 (4.41)	−0.21	0.83	0.03	**−0.26** **(*p* = 0.0001)**	+0.02 (*p* = 0.71)	18.22 (4.73)	17.84 (4.19)	0.59	0.56	−0.08	**19.10 (4.46)**	**16.95 (4.51)**	**17.35 (4.48)**	**7.22**	**0.001**	**0.12**
**BPS**	27.39 (7.51)	26.53 (6.89)	28.15 (7.97)	−1.74	0.08	0.22	−0.16 (*p* = 0.013)	−0.09 (*p* = 0.14)	27.24 (7.55)	27.82 (7.45)	−0.55	0.58	0.08	27.56 (7.75)	27.06 (7.15)	28.55 (8.56)	0.27	0.77	0.002

**Table 2:** Pearson’s correlation coefficients (*r*) are reported controlling for gender, age, education level, and marital status. In the table, *p* = 0.0001, *p* = 0.001, and *p* < 0.006 are indicated by *, °, and ** respectively.

	MSQ-Sleep	MSQ-Wake	WMPoS	FMPoS	Disengagement	High Arousal	Low Arousal	Inattention	Time Perception	MSBS	MAS	BPS
MSQ-sleep	1	+0.67 *	+0.14	−0.02	+0.37 *	+0.37 *	+0.35 *	+0.35 *	+0.33 *	+0.42 *	+0.32 *	+0.29 *
MSQ-wake	-	1	+0.20 °	+0.10	+0.51 *	+0.47 *	+0.48 *	+0.47 *	+0.31 *	+0.53 *	+0.41 *	+0.43 *
WMPoS	-	-	1	+0.58 *	+0.14	+0.20 °	+0.12	+0.25 *	+0.06	+0.18 **	+0.17 **	+0.20 °
FMPoS	-	-	-	1	+0.10	+0.13	+0.03	+0.17 **	−0.02	+0.10	+0.11	+0.30 *
Disengagement	-	-	-	-	1	+0.81 *	+0.82 *	+0.76 *	+0.50 *	+0.92 *	+0.48 *	+0.27 *
High Arousal	-	-	-	-	-	1	+0.81 *	+0.74 *	+0.45 *	+0.91 *	+0.40 *	+0.23 *
Low Arousal	-	-	-	-	-	-	1	+0.65 *	+0.46 *	+0.89 *	+0.39 *	+0.18 **
Inattention	-	-	-	-	-	-	-	1	+0.35 *	+0.83 *	+0.48 *	+0.33 *
Time Perception	-	-	-	-	-	-	-	-	1	+0.65 *	+0.26 *	+0.05
MSBS	-	-	-	-	-	-	-	-	-	1	+0.48 *	+0.25 *
MAS	-	-	-	-	-	-	-	-	-	-	1	+0.23 *
BPS	-	-	-	-	-	-	-	-	-	-	-	1

**Table 3:** The table presents the results of the mediation models, including effects (*β*), standard errors (SE), *t* and *p* values, and 95% CIs (low and up limits) for predictors, mediators, and covariates. Additionally, R^2^, *F* statistics, and overall *p* values are reported for each model, along with direct and indirect effects. Note that the statistics for the paths MSBS → MAS, MSBS → BPS, and MAS → BPS (including covariates) are identical across all models.

	*β*	SE	*t*	*p*	95-CI Low Limit	95-CI Up Limit	*R* ^2^	*F*	*p*
(1a) Mediation Model of the relationship between MSBS and MSQ-sleep Outcome variable: MAS							0.28	*F*(5,253) = 19.54	0.00001
MSBS → MAS	+0.33	0.04	+8.47	0.00001	+0.25	+0.41			
Covariate: Gender	−0.03	0.25	−0.11	0.92	−0.51	+0.46			
Covariate: Age	−0.05	0.02	−2.56	0.011	−0.09	−0.01			
Covariate: Education	+0.15	0.19	+0.76	0.45	−0.23	+0.52			
Covariate: Marital Status	+0.15	0.24	+0.61	0.54	−0.33	+0.62			
(1b) Mediation Model of the relationship between MSBS and MSQ-sleep Outcome variable: BPS							0.34	*F*(6,252) = 5.67	0.00001
MSBS → BPS	+0.22	0.08	+2.75	0.006	+0.06	+0.38			
MAS → BPS	+0.23	0.11	+1.99	0.048	+0.002	+0.45			
Covariate: Gender	+0.16	0.45	+0.35	0.72	−0.72	+1.04			
Covariate: Age	−0.08	0.04	−2.22	0.03	−0.15	−0.009			
Covariate: Education	−0.49	0.35	−1.41	0.16	−1.18	+0.19			
Covariate: Marital Status	+1.20	0.44	+2.74	0.007	+0.34	+2.07			
(1c) Mediation Model oft he relationship between MSBS and MSQ-sleep Outcome variable: MSQ-sleep							0.25	*F*(7,251) = 11.89	0.0001
MSBS → MSQ-sleep	+0.30	0.06	+4.97	0.00001	+0.18	+0.41			
MAS → MSQ-sleep	+0.17	0.08	+1.99	0.04	+0.001	+0.33			
BPS → MSQ-sleep	+0.15	0.05	+3.33	0.001	+0.06	+0.24			
Covariate: Gender	−0.64	0.33	−1.94	0.053	−1.28	+0.009			
Covariate: Age	+0.03	0.03	+1.24	0.22	−0.02	+0.08			
Covariate: Education	−0.42	0.26	−1.64	0.10	−0.92	+0.08			
Covariate: Marital Status	+0.11	0.33	+0.34	0.74	−0.53	+0.75			
(1d) Direct Effect: MSBS → MSQ-sleep	+0.29	0.06	+4.97	0.00001	+0.17	+0.41			
(1d) Indirect Effect: MSBS → MAS → MSQ-sleep	+0.06	0.03			−0.003	+0.12			
(1d) Indirect Effect: MSBS → BPS → MSQ-sleep	+0.03	0.02			+0.008	+0.07			
(1d) Indirect Effect: MSBS → MAS → BPS → MSQ-sleep	+0.01	0.007			+0.00001	+0.03			
(2a) Mediation Model of the relationship between MSBS and MSQ-wake Outcome variable: MAS							0.28	*F*(5,253) = 19.54	0.00001
MSBS → MAS	+0.33	0.04	+8.47	0.00001	+0.25	+0.41			
Covariate: Gender	−0.03	0.25	−0.11	0.92	−0.51	+0.46			
Covariate: Age	−0.05	0.02	−2.56	0.011	−0.09	−0.01			
Covariate: Education	+0.15	0.19	+0.76	0.45	−0.23	+0.52			
Covariate: Marital Status	+0.15	0.24	+0.61	0.54	−0.33	+0.62			
(2b) Mediation Model of the relationship between MSBS and MSQ-wake Outcome variable: BPS							0.34	*F*(6,252) = 5.67	0.00001
MSBS → BPS	+0.22	0.08	+2.75	0.006	+0.06	+0.38			
MAS → BPS	+0.23	0.11	+1.99	0.048	+0.002	+0.45			
Covariate: Gender	+0.16	0.45	+0.35	0.72	−0.72	+1.04			
Covariate: Age	−0.08	0.04	−2.22	0.03	−0.15	−0.009			
Covariate: Education	−0.49	0.35	−1.41	0.16	−1.18	+0.19			
Covariate: Marital Status	+1.20	0.44	+2.74	0.007	+0.34	+2.07			
(2c) Mediation Model of the relationship between MSBS and MSQ-wake Outcome variable: MSQ-wake							0.49	*F*(7,251) = 33.97	0.00001
MSBS → MSQ-wake	+0.30	0.04	+7.08	0.00001	+0.22	+0.38			
MAS → MSQ-wake	+0.16	0.06	+2.70	+0.007	+0.04	+0.28			
BPS → MSQ-wake	+0.19	0.03	5.87	0.00001	+0.13	+0.26			
Covariate: Gender	−0.33	0.23	−1.43	0.15	−0.79	+0.12			
Covariate: Age	−0.07	0.02	−3.96	0.0001	−0.11	−0.04			
Covariate: Education	−0.35	0.18	−1.91	0.06	−0.71	+0.01			
Covariate: Marital Status	+0.44	0.23	1.90	0.06	−0.02	+0.90			
(2d) Direct Effect: MSBS → MSQ-wake	+0.30	0.04	+7.08	0.00001	+0.22	+0.38			
(2d) Indirect Effect: MSBS → MAS → MSQ-wake	+0.05	0.02			+0.009	+0.10			
(2d) Indirect Effect: MSBS → BPS → MSQ-wake	+0.04	0.02			+0.01	+0.08			
(2d) Indirect Effect MSBS → MAS → BPS → MSQ-wake	+0.01	0.008			+0.00001	+0.03			
(3a) Mediation Model of the relationship between MSBS and WMPoS Outcome variable: MAS							0.28	*F*(5,253) = 19.54	0.00001
MSBS → MAS	+0.33	0.04	+8.47	0.00001	+0.25	+0.41			
Covariate: Gender	−0.03	0.25	−0.11	0.92	−0.51	+0.46			
Covariate: Age	−0.05	0.02	−2.56	0.011	−0.09	−0.01			
Covariate: Education	+0.15	0.19	+0.76	0.45	−0.23	+0.52			
Covariate: Marital Status	+0.15	0.24	+0.61	0.54	−0.33	+0.62			
(3b) Mediation Model of the relationship between MSBS and WMPoS Outcome variable: BPS							0.34	*F*(6,252) = 5.67	0.00001
MSBS → BPS	+0.22	0.08	+2.75	0.006	+0.06	+0.38			
MAS → BPS	+0.23	0.11	+1.99	0.048	+0.002	+0.45			
Covariate: Gender	+0.16	0.45	+0.35	0.72	−0.72	+1.04			
Covariate: Age	−0.08	0.04	−2.22	0.03	−0.15	−0.009			
Covariate: Education	−0.49	0.35	−1.41	0.16	−1.18	+0.19			
Covariate: Marital Status	+1.20	0.44	+2.74	0.007	+0.34	+2.07			
(3c) Mediation Model of the relationship between MSBS and WMPoS Outcome: WMPoS							0.29	*F*(7,251) = 14.89	0.00001
MSBS → WMPoS	+0.01	0.01	+0.95	0.34	−0.01	+0.03			
MAS → WMPoS	+0.02	0.02	+1.004	0.32	−0.02	+0.05			
BPS → WMPoS	+0.05	0.009	+5.51	0.00001	+0.03	+0.06			
Covariate: Gender	+0.04	0.06	+0.72	0.47	−0.08	+0.16			
Covariate: Age	−0.01	0.005	−2.26	0.02	−0.02	−0.001			
Covariate: Education	−0.12	0.05	−2.62	0.009	−0.22	−0.03			
Covariate: Marital Status	−0.18	0.06	−2.95	0.004	−0.30	−0.06			
(3d) Direct Effect: MSBS → WMPoS	+0.01	0.01	+0.95	0.34	−0.01	+0.03			
(3d) Indirect Effect: MSBS → MAS → WMPoS	+0.005	0.005			−0.005	+0.02			
(3d) Indirect Effect: MSBS → BPS → WMPoS	+0.01	0.004			+0.003	+0.02			
(3d) Indirect Effect: MSBS → MAS → BPS → WMPoS	+0.004	0.002			+0.0001	+0.008			
(4a) Mediation Model of the relationship between MSBS and FMPoS Outcome variable: MAS							0.28	*F*(5,253) = 19.54	0.00001
MSBS → MAS	+0.33	0.04	+8.47	0.00001	+0.25	+0.41			
Covariate: Gender	−0.03	0.25	−0.11	0.92	−0.51	+0.46			
Covariate: Age	−0.05	0.02	−2.56	0.011	−0.09	−0.01			
Covariate: Education	+0.15	0.19	+0.76	0.45	−0.23	+0.52			
Covariate: Marital Status	+0.15	0.24	+0.61	0.54	−0.33	+0.62			
(4b) Mediation Model of the relationship between MSBS and FMPoS Outcome variable: BPS							0.34	*F*(6,252) = 5.67	0.00001
MSBS → BPS	+0.22	0.08	+2.75	0.006	+0.06	+0.38			
MAS → BPS	+0.23	0.11	+1.99	0.048	+0.002	+0.45			
Covariate: Gender	+0.16	0.45	+0.35	0.72	−0.72	+1.04			
Covariate: Age	−0.08	0.04	−2.22	0.03	−0.15	−0.009			
Covariate: Education	−0.49	0.35	−1.41	0.16	−1.18	+0.19			
Covariate: Marital Status	+1.20	0.44	+2.74	0.007	+0.34	+2.07			
(4c) Mediation Model of the relationship between MSBS and FMPoS Outcome variable: FMPoS							0.40	*F*(7,251) = 23.60	0.00001
MSBS → FMPoS	+0.002	0.001	+0.16	0.88	−0.02	+0.03			
MAS → FMPoS	+0.01	0.02	+0.59	0.56	−0.02	+0.04			
BPS → FMPoS	+0.04	0.009	+4.67	0.00001	+0.03	+0.06			
Covariate: Gender	+0.06	0.07	+0.93	0.35	−0.07	+0.19			
Covariate: Age	−0.04	0.005	−6.75	0.00001	−0.05	−0.03			
Covariate: Education	−0.07	0.05	−1.30	0.19	−0.17	+0.03			
Covariate: Marital Status	−0.19	0.07	−2.92	0.004	−0.32	−0.03			
(4d) Direct Effect: MSBS → FMPoS	+0.002	0.01	+0.16	0.88	−0.02	+0.03			
(4d) Indirect Effect: MSBS → MAS → FMPoS	+0.003	0.006			−0.009	+0.02			
(4d) Indirect Effect: MSBS → BPS → FMPoS	+0.0096	0.004			+0.003	+0.02			
(4d) Indirect Effect: MSBS → MAS → BPS → FMPoS	+0.003	0.002			+0.0001	+0.007			

## Data Availability

The raw data supporting the conclusion of this article will be made available by the corresponding author upon request.
